# Methods for the Destruction of Flies and Mosquitoes

**Published:** 1919-01

**Authors:** 


### HYGIENE

---

**Methods for the Destruction of Flies and Mosquitoes.** By Merchants' Association of New York. *The Military Surgeon*, August, 1918.

The Merchants' Association of New York recently published a leaflet urging the destruction of flies. A series of recipes and preventives against them are then given.

The U. S. Government suggests formaldehyde and sodium salicylate as the two best fly poisons. Formaldehyde or sodium salicylate solution is made by adding 3 teaspoonfuls of the pure chemical to a pint of water. An ordinary drinking glass is filled with the solution and quickly inverted on a saucer, on which a piece of white blotting paper is placed. A match is fitted under the saucer and the container is ready for use. Preventives, such as lavender, geranium, white clover, etc., are recommended. According to a French scientist, rooms decorated in blue help to keep out the flies. Pyrethrum powder may also be burned, but it only stupefies the flies which must be swept and burned. Borax is valuable around farms and out of doors; lye, chloride of lime or copperas dissolved in water, crude carbolic acid, etc., may also be used.

In connection with army buildings, Major I. W. Brewer, M. R. C., makes the following suggestions :

In addition to the doors leading to the kitchens, each mess hall should be provided with doors situated at the end farthest from the kitchen and opening into a vestibule kept as dark as possible. The doors leading from the vestibule into the mess hall should be provided with screens and those opening into the outside made of solid material.

Alimo cookers might be prevented from smoking by constructing special supports for the upper part of the alimo.

Latrines should be provided with ventilators in order to prevent moisture from consolidating on the lids of the seats of Havard boxes. Fly traps have, in some cases, been placed on a board just fitting into the hole of the latrine box.

The camp latrines at present in use are very unsatisfactory owing to the length of piping connecting them with the latrine pit. A simple urinal, with a minimum amount of piping, was devised last summer at Fort Ethan Allen.

Lieut. P. H. Brigham, M. R. C., invented two devices for disposing of mosquitoes. The automatic oiling device consists of a bottle filled with crude oil, closed up by means of a cork with 2 grooves, one on each side. A little oil is poured into one groove and the bottle submerged in a nearly upright position. The water, percolating through the second groove, sinks to the bottom of the bottle and displaces the oil, which runs through the oil groove and floats on the surface of the water, covering large areas. Drops of oil are thrown out every 5 minutes and one bottle lasts approximately 3 days. In one instance, an automatic oiling bottle was provided with a small parachute and shot over a pond with a bow gun.

The mosquito electrocutor consists of a wooden frame enclosing a screen of positive and negative copper wires placed alternately. The minimum voltage required is 110 volts.

